# Genetic analysis of clinical findings at health examinations of young Swedish warmblood riding horses

**DOI:** 10.1186/1751-0147-55-22

**Published:** 2013-03-08

**Authors:** Lina Jönsson, Anna Näsholm, Lars Roepstorff, Agneta Egenvall, Göran Dalin, Jan Philipsson

**Affiliations:** 1 Department of Animal Breeding and Genetics, Swedish University of Agricultural Sciences, PO Box 7023, Uppsala 750 07, Sweden; 2 Department of Equine Studies, Swedish University of Agricultural Sciences, PO Box 7043, Uppsala 750 07, Sweden; 3 Department of Clinical Sciences, Swedish University of Agricultural Sciences, PO Box 7054, Uppsala 750 07, Sweden

**Keywords:** Medical health, Orthopaedic health, Flexion test, Hoof, Clinical signs, Heritability, Genetic correlation

## Abstract

**Background:**

Soundness is important for welfare and utility of the riding horse. Musculoskeletal disorders are the most common causes of interruption in training and of culling. Despite great importance, heritability of a majority of health traits in horses has previously not been estimated. The objective was to perform genetic analyses of medical and orthopaedic health traits in young riding horses, including estimates of heritability and genetic correlations between health traits, and to reveal possibilities for genetic evaluation of stallions for progeny health.

**Results:**

The heritability of health traits was estimated using records from 8,238 Swedish warmblood riding horses examined as 4–5 year olds at the Riding Horse Quality Test in 1983–2005. The analyses were performed using multi-trait linear mixed animal models. The heritabilities of palpatory orthopaedic health (PALP), including effusion, swelling, heat, soreness and stiffness/atrophy, and hoof examination results (HOOF), of hoof shape and hoof wall quality, were 0.12 and 0.10, respectively. The genetic variation in these traits resulted in distinct health differences between progeny groups of stallions. The highest heritability among clinical signs of PALP was found for synovial effusions at 0.14. For systemic locations, joint related findings had the highest heritability; 0.13. The heritabilities of medical health and locomotion examination results were low, 0.02 and 0.04, respectively. A genetic improvement of health status has occurred over time but accounts only partly for the decrease in clinical findings of health during the studied period.

**Conclusions:**

The genetic variation found in PALP and HOOF implies distinct differences between progeny groups. Thus, there are possibilities for improvement of these traits in the population through selection. The weak and non-significant correlation between PALP and HOOF suggests that both traits need to be selected for in practical breeding to improve both traits. Some genetic improvements over time have already been achieved, possibly due to regular stallion health inspections and an indirect selection for lifetime performance. For further improvements stallion breeding values for health may be introduced, based on RHQT examinations, complementary to present breeding values for performance.

## Introduction

Health status plays a major role for the longevity of the horse. Musculoskeletal disorders are the most common reasons for culling of riding horses [1-4]. In order to improve animal welfare and longevity it is important to increase the knowledge of underlying causative factors and to develop further strategies to reduce health disorders. Health of the horse is to a large extent dependent on what trauma, physical strains and other environmental stresses it has been exposed to. However, a genetic predisposition to stay sound might be present, whereby some horses endure environmental loads and strains better than others. Further, inherited predispositions, e.g. for osteochondrosis (OC), bone spavin, and navicular disease, can influence the health of the horse [5-7]. Several European breeding organisations consider health to be an important aspect in breeding (14 out of 19) [8]. In the Swedish Warmblood breed (SWB) soundness, defined as durability of the horse, is included in the stated breeding goal with an intention for health traits to be part of the genetic evaluation and breeding scheme [8]. However, knowledge about the genetic background of health traits is scarce due to a lack of systematic health recording of normal horse populations. Such recordings need to be developed as basis for further preventive actions.

A unique source of health information is available from the Riding Horse Quality Test (RHQT) in Sweden, where 4-year-old horses of both genders and 5-year-old mares that had a foal the previous year, have been examined and recorded for health status and performance since 1973. All broken-in young riding horses can participate regardless of talent. If assuming 7% loss each year due to death, export etc. [1] approximately 23% of available 4-year-olds are tested. Test locations are distributed throughout the whole country. The overall score of orthopaedic health of 1,815 horses has earlier shown to be significantly correlated to future risk of culling [9] and to have a heritability of 0.06 (n = 3,708) [10]. In a more recent study, the locomotion examination (LOCO) was shown to influence the overall veterinary score of orthopaedic health to a high degree, as did palpatory orthopaedic health (PALP) [11]. Palpatory findings were predominantly located to joints, and effusion was the most frequent clinical sign. Acute clinical findings, defined as in [11] had a large individual effect on the overall orthopaedic health score, but were generally rare in the studied population, whereas chronic findings were more common but generally had lower effects on the overall health score. However, also the heritability of the health traits needed elucidation.

Thus, the aim of the current study was to perform genetic analysis of medical and orthopaedic health traits in young riding horses participating in the RHQT, to estimate heritabilities and genetic correlations between health traits, and to reveal possibilities for genetic evaluation of stallions for health traits in their progeny.

## Material and methods

### Material

Health data were obtained for Swedish warmblood riding horses participating in the RHQT in 1983–2005, except for horses examined in 1985–1987 (due to loss of data). When applying for participation in the RHQT horse owners gave their consent to use the results in research. Health inspections of all horses at an event were performed in the same environment, by one veterinarian for health examination 1, and another veterinarian for health examination 2 under the same conditions. For types of examination, see Table 1 and [11] for further details including protocols for the examinations. Health examination 1 included a medical health examination (MED) of e.g. mucous membranes, skin, mouth, lungs and heart, including 28 traits scored between 0 and 3 (0: no clinical finding, 1: minor, 2: moderate or 3 severe clinical finding), and a hoof examination (HOOF) for hoof shape and quality, including 11 traits scored 0–3. Health examination 2 included a palpatory orthopaedic health examination (PALP), i.e. recording and evaluation of effusion, swelling, soreness, heat or stiffness/atrophy in separate anatomic locations of each limb, including 324 traits scored 0–3. It also included a locomotion examination (LOCO) in walk and trot, and trot after flexion test for each limb, including 12 traits scored 0–3. An overall assessment score for health examination 1 and 2 (H1 and H2, respectively) between 1 (very poor) and 10 (excellent) was given by the examining veterinarian. During the studied years, all horses were documented for the same health traits in identical protocols. Examiners were experienced horse practitioners with compulsory training in judging at the RHQT.

**Table 1:** Health traits and scales of scoring health examination results at the Riding Horse Quality Test (RHQT)

	**No of traits**	**Scale of scoring each trait**
**Medical health examination (MED)**		
Examination of body, skin, mucous membranes, mouth etc. including auscultation of heart and lungs	28	0–3
**Hoof examination (HOOF)**		
Examination of hoof shape and quality	11	0–3
**Palpatory orthopaedic examination (PALP)**		
Palpation for effusion, swelling, soreness, heat, stiffness and/or atrophy, respectively at^1^:	5 clinical signs at	0–3
Locations in forelimbs/shoulder	18 * 2(left/right)	0–3
Locations in hindlimbs/croup	23 * 2(left/right)	0–3
**Locomotion examination (LOCO)**		
Unprovoked movements in walk, each limb	4	0–3
Unprovoked movements in trot, each limb	4	0–3
Flexion test followed by trot, each limb	4	0–3
**Overall assessment scores**		
Overall score for medical health including hoof quality (H1)	1	1–10
Overall score for orthopaedic health including flexion test (H2)	1	1–10

^1^If applicable at location.

0–3 scale: 0 = no finding, 1 = minor finding, 2 = moderate finding, 3 = severe finding.

1–10 scale; 10–9 = excellent health, 8 = good health, 7–6 = acceptable health, 5–4 = minor/moderate signs of unsoundness/injury, 3 = not in show condition, 2–1 = severe unsoundness/injury.

The identity of each horse was confirmed in the SWB pedigree database. The study included 8,238 horses examined at 187 events with ≥ 10 participating horses at each, from 602 sires of the SWB population. The 602 stallions were represented with between 1 and 189 tested progeny each, where 133 stallions had at least 20 tested progeny. The pedigree information of studied horses and their ancestors included 29,693 horses. For further descriptive statistics see [11].

### Methods

**Figure 1:**
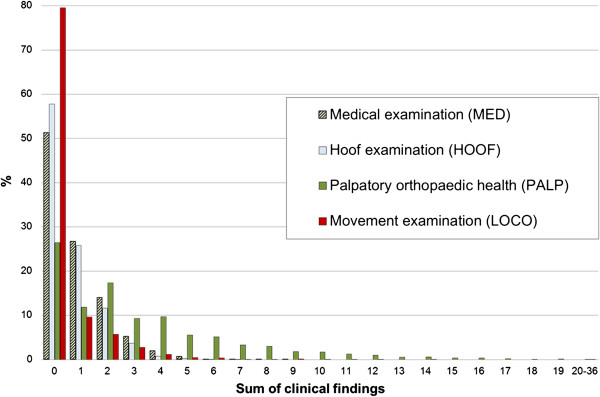
**Distribution of sum of clinical findings, including severity, for each type of examination.** Results include 28 traits within MED, 11 traits within HOOF, 324 traits within PALP and 12 traits within LOCO, all separately scored 0–3. The x-axis represents the sum of clinical findings and the y-axis the percent of studied horses within each group of summed clinical findings, N = 8,212–8,238 examined in years 1983–2005.

Health traits were grouped according to type of examination, i.e. MED, HOOF, PALP and LOCO, respectively. Each group represents the summed value of number and severity (0–3) of clinical findings for each horse within type of examination. Thus, the sum of clinical findings increased both with number and severity of findings (see Figure 1 for distribution). Summed values include bilateral findings. Corresponding summation of PALP findings were performed in 4 groups of systemic location, i.e. joints, muscles, tendons & suspensory ligaments and skeleton & hoof cartilage, and 5 groups of clinical signs, i.e. effusion, heat, soreness, swelling, stiffness/atrophy, where bilateral findings were not accounted for. The heritability of each of these groups, i.e. groups of type of examination and overall scores, clinical signs, and systemic locations, respectively, were estimated from 3 multi-trait mixed animal model analyses. In addition, heritabilities for separate health traits were estimated by groups in multi-trait analyses if the prevalence was ≥ 3% [11]. Including traits with lower prevalence resulted in unreliable parameter estimates. Complementary bivariate analysis including sum of findings for effusions and joint related traits, respectively, were also performed. Restricted maximum likelihood (REML) estimation was used, executed by an average information algorithm in DMU^a^. In the same analysis Best Linear Unbiased Prediction (BLUP) breeding values for each animal was produced.

According to initial analyses of variance, significant effects of event (p < 0.0001) and gender (p < 0.05) were present in analyses of all types of examinations, except for hooves, where no significant gender difference was found [11]. The age effects were generally non-significant and were excluded in the final analyses, which included only event and gender as fixed effects. Thus, the applied model for the genetic analyses was:

$$y_{\mathrm{ijk}}=\mu +\mathrm{even}t_{i}+\mathrm{gende}r_{j}+\mathrm{anima}l_{k}+e_{\mathrm{ijk}}$$

where *y*_*ijk*_ is the score of each trait for the *k*^*th*^ horse, *μ* is the population mean; *event*_*i*_ is the fixed effect of the *i*^*th*^ event, representing the combined effect of place, year and veterinarian; *gender*_*j*_ is the fixed effect of gender (female or male); *animal*_*k*_ is the random genetic effect of the *k*^*th*^ horse, and *e*_*ijk*_ is the random residual effect. An identical model, except that event was analysed as a random effect, was also applied to obtain measures of the event variation in PALP and HOOF, respectively.

Untransformed variables were used in subsequent analyses because extended transformation trials showed similar heritability estimates and deviations from normal distribution of residuals compared to untransformed data. Estimates of standard errors of phenotypic correlations were obtained with Taylor series expansion [12] using components produced by DMU. Coefficients of determination (R^2^) for analyses of separate health traits were derived from General Linear Model (GLM) analyses in SAS^b^, including fixed effects of event and gender. The additive genetic coefficients of variation (CV) were calculated to estimate the genetic variation of PALP, HOOF, LOCO as:

$$\mathrm{CV}=\frac{\sqrt{\mathrm{genetic}\;\mathrm{variance}}}{\mathrm{trait}\;\mathrm{mean}}*100$$

### Presentation of breeding values

Distribution of estimated breeding values (EBVs) and genetic trends for PALP and HOOF were produced. In order to use a scale common in routine genetic evaluations the EBVs were transformed to a mean of 100 and a genetic standard deviation of 20. The 8,238 examined horses were used as reference population (mean EBV = 100) for calculations of the relative breeding values. The highest breeding values represent the best ability to produce healthy offspring. Reliability of estimated breeding values was calculated as below, where PEV = predicted error variance:

$$\mathrm{Reliability}=1-\frac{\mathrm{PEV}}{\mathrm{genetic}\;\mathrm{variane}}$$

Genetic trends were calculated for horses born in 1983–2001, as no or few examined horses born before 1983 were available. Due to unreliable deviations from the overall trend in the first and last year of the period, the trend per year was calculated by fitting a regression line to the trend for the years 1984–2000. The overall progress in 1983–2001 was calculated by extrapolation of the annual progress.

## Results

### Heritability

Estimated heritabilities of overall scores and sum of clinical findings are presented in Table 2 along with mean and 90^th^ percentile of analysed traits. The heritabilities for overall scores were low (0.04–0.06), whereas the highest estimates were found within separate types of examination (0.10–0.14). Estimates for combined results of several types of examination (PALP, LOCO, HOOF and MED) generated slightly lower heritabilities for each new type added (results not shown), suggesting that different types of examinations represent traits with different genetic background.

**Table 2:** **Estimated heritabilities (h**^**2**^**) with standard errors (s.e.), accompanied by means and 90**^**th **^**percentiles**

	**Mean**	**90**^**th **^**percentile**	**h**^**2**^	**s.e.**
**Overall health scores**				
Health examination 1 (H1)	9.42	10	0.04	0.01
Health examination 2 (H2)	8.79	10	0.06	0.02
**Overall health within type of examination**				
Medical examination (MED)	0.83	2	0.02	0.01
Hoof examination (HOOF)	0.65	2	0.10	0.02
Palpatory orthopaedic examination (PALP)	3.21	8	0.12	0.02
Locomotion examination (LOCO)	0.42	2	0.04	0.01
**Clinical signs of PALP findings**				
Effusion	1.38	4	0.14	0.02
Heat	0.01	0	0.00	0.01
Soreness	0.06	0	0.03	0.01
Swelling	0.68	2	0.06	0.02
Stiffness/Atrophy	0.10	0	0.02	0.01
**Systemic location of PALP findings**				
Tendons & Ligaments	0.03	0	0.00	0.01
Muscles	0.15	1	0.04	0.01
Joints	1.61	4	0.13	0.02
Skeleton and hoof cartilage	0.45	2	0.04	0.01

Included traits representing overall health scores and summed clinical findings, including severity, by type of examination. Within palpatory orthopaedic health (PALP) estimates of clinical signs-, and systemic locations were also performed. (N = 8,212–8,238).

### HOOF and MED examinations

The heritability of summed hoof findings (HOOF) was estimated at 0.10 (Table 2). Within HOOF, separate traits with a prevalence ≥ 3% and a heritability of at least 0.05, (not shown in table) were found for flat hooves at 0.11, small hooves at 0.09, hoof wall cracks at 0.07, and contracted heels at 0.07, that all had a s.e. of 0.02. For MED, the heritability of summed findings was estimated at 0.02. Three separate MED traits with prevalence over 3% obtained a heritability ≥0.05: General clinical mouth findings (overrepresented by wolf teeth) at 0.09, parrot mouth at 0.09, and skin sarcoids at 0.05, all with s.e. of 0.01–0.02.

### Palpatory orthopaedic health (PALP)

Summed findings of PALP showed a heritability of 0.12 compared to the heritability of the H2 score at 0.06, where PALP traits otherwise are included (Table 2). Seven separate PALP traits with ≥ 3% prevalence and a heritability of ≥0.05 (not shown in table) were found for effusions in tarsocrural joints (0.09), hindlimb digital flexor tendon sheaths (0.09), middle/lower part of carpal joints (0.07), forelimb distal interphalengeal (coffin) joints (0.07), metatarsophalangeal (hindlimb fetlock) joints (0.06), metacarpophalangeal (forelimb fetlock) joints (0.06) and swelling in the proximal part of metacarpus (0.05), all with a s.e. of 0.01–0.02. Estimates for groups of clinical signs indicated that effusions are distinctly more dependent on a genetic background (h^2^ = 0.14) than other clinical signs (h^2^ = 0.00–0.06). Further, joint related findings were more affected by inheritance (h^2^ = 0.13) than other systemic locations (h^2^ = 0.00–0.04).

### Locomotion examination (LOCO)

The heritability of summed LOCO findings including flexion test reactions was estimated at 0.04 which may be compared to the H2 score estimate at 0.06, where locomotion examination results were incorporated (Table 2). The R^2^ value of LOCO was lower (0.15) compared to other performed examinations (MED, PALP and HOOF) where 0.31–0.37 of the variation was explained by fixed effects. The heritability of separate fore- and hindlimb flexion test reactions, respectively, suggested forelimb reactions to be more heritable (0.06, s.e. 0.02) than hindlimb reactions (0.01, s.e.0.01). R^2^ values indicated forelimb flexion test results to be less influenced by included fixed effects (0.12) compared to hindlimb results (0.16). The prevalence of unprovoked lameness in fore- and hindlimbs, respectively, was too small for further evaluation (<3%).

### Correlations

Strong significant genetic correlations (Table 3) showed that a high sum of LOCO and PALP findings usually were found with lower H2 scores and vice versa (−0.82 and −0.76, respectively). Thus, a high breeding value for H2 was favourably correlated to good breeding values of LOCO and PALP, where selection for one trait will also improve the other health trait. The genetic correlation between LOCO and PALP was 0.40. A strong correlation between the H1 score and HOOF findings was found at −0.86. No correlation between MED and HOOF was found, and a non-significant correlation of −0.22 was found between the H1 score and MED. The correlation between HOOF and PALP was non-significant at 0.11. Corresponding phenotypic correlations showed similar trends, although usually at lower absolute levels.

**Table 3:** Genetic correlations above diagonal and phenotypic correlations below diagonal, with standard error in subscript

	** H1**	** H2**	** MED**	** HOOF**	** PALP**	** LOCO**
Overall health score of health examination 1 (H1)		0.13 _0.20_	−0.22 _0.28_	−0.86 _0.10_	−0.08 _0.17_	−0.08 _0.24_
Overall health score of health examination 2 (H2)	0.08 _0.02_		−0.44 _0.26_	−0.25 _0.16_	−0.76 _0.08_	−0.82 _0.09_
Medical examination (MED)	−0.42 _0.02_	−0.05 _0.01_		0.00 _0.25_	0.22 _0.22_	0.56 _0.32_
Hoof examination (HOOF)	−0.44 _0.02_	−0.03 _0.01_	0.02 _0.01_		0.11 _0.14_	0.27 _0.19_
Palpatory orthopaedic examination (PALP)	−0.06 _0.09_	−0.48 _0.10_	0.03 _0.09_	0.04 _0.09_		0.40 _0.16_
Locomotion examination (LOCO)	−0.05 _0.01_	−0.63 _0.02_	0.03 _0.01_	0.02 _0.00_	0.25 _0.01_	

Included traits representing overall health scores and sum of clinical findings within type of examination.

Medium to high positive genetic correlations of 0.18–0.67 (s.e. 0.11–0.17), were found between all combinations of effusions in middle/lower part of carpal joint, tarsocrural joint, fore- and hindlimb fetlocks and hindlimb digital flexor tendon sheaths. In total, 8 of 10 previously mentioned correlations were significant, suggesting these traits to have partly the same genetic background (not shown in table). Additional analyses also showed that the genetic correlation between effusions and palpatory joint findings were very high with an estimated value of 1.00 (s.e. 0.01) and a phenotypic correlation of 0.92, thus most effusions are found in joints and most joint findings are effusions, and these traits could be considered to have the same genetic background. As regards hoof correlations small hooves and contracted heels were significantly related (r_g_ = 0.31, s.e. 0.15).

### Estimated breeding values (EBVs) for PALP and HOOF-traits with highest heritability

**Figure 2:**
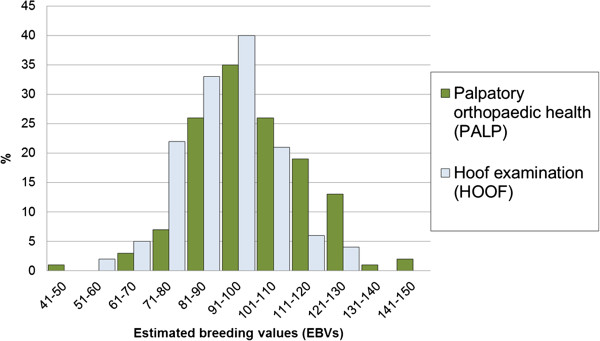
**Distribution of breeding values (EBVs) of palpatory orthopaedic health (PALP) and hoof examination findings (HOOF).** Estimates are transformed to a scale with 100 as mean and a genetic standard deviation of 20. High breeding values represent stallions with the healthiest offspring. Stallions with at least 20 progeny are included (N = 133 stallions).

The additive genetic coefficients of variation showed HOOF and PALP to have the largest genetic variation of 36.9% and 30.4%, respectively. Further, LOCO also had a considerable variation of 9.1%. The distribution of EBVs for PALP and HOOF findings (Figure 2) indicated a normal distribution among 133 stallions with at least 20 examined offspring. A large variation among offspring of stallions was demonstrated. The top 3 stallions according to breeding values for PALP produced 14–32% units more progenies free from clinical PALP findings compared to the 3 stallions with lowest breeding values. Further, the top 3 stallions according to HOOF breeding values produced 26–56% units more progeny without clinical hoof findings compared to the 3 stallions with lowest HOOF breeding values. For HOOFs the reliability of EBVs of stallions with 20 offspring was 42%, and among all stallions with at least 20 offspring it was 58%. This could be compared to mares with 2 examined offspring having a reliability of 24%. Corresponding results for PALP were 43% for stallions with 20 offspring, 59% among all stallions with at least 20 offspring, and 23% for mares with 2 examined offspring, respectively. Thus, the difference in reliability was almost twice as high for stallions with 20 offspring compared to mares with 2 offspring.

### Genetic trend of PALP and HOOF of examined horses

**Figure 3:**
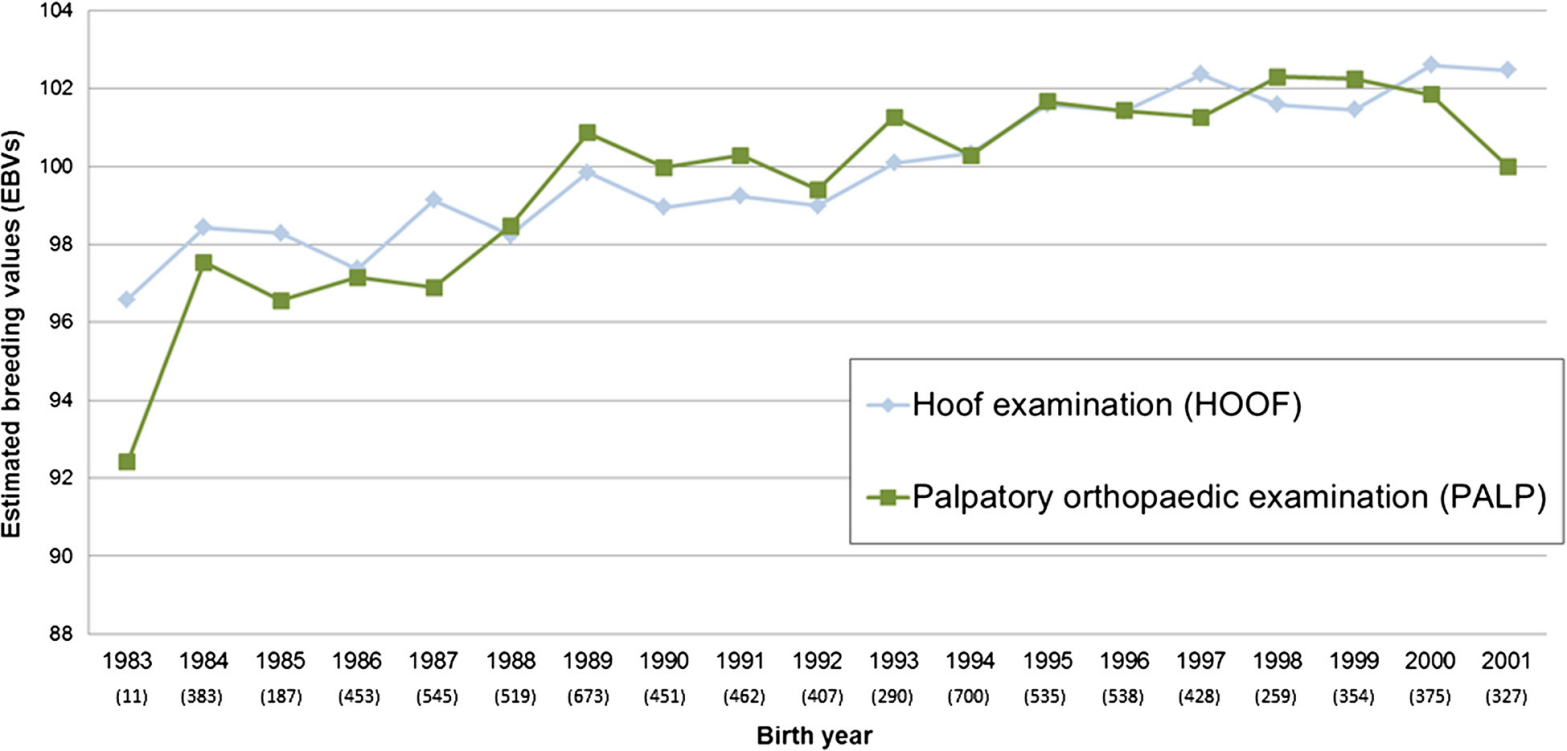
**Genetic trend of palpatory orthopaedic health (PALP) and hoof findings (HOOF) in examined horses born in 1983–2001.** Estimates are presented on a scale of 100 as mean, where all examined horses (N = 8,238) constitute the reference population. Number of examined horses each birth year is presented within brackets.

The trends indicated a slight genetic improvement of 0.26 and 0.32 genetic standard deviations for HOOF and PALP, respectively, for the whole period 1983–2001 (Figure 3). This corresponds to an annual improvement of 0.01–0.02 genetic standard deviations. The trends were quite stable over years with a large number of available horses each birth year (n = 187–700), except for years 1983 and 2001.

The variance component for event as a random effect was estimated at 2.27 for PALP and at 0.25 for HOOF with a decrease in the prevalence of findings throughout the study period. The genetic variance component was estimated at 0.46 for PALP and at 0.06 for HOOF. Thus, the effect of event was 4–5 times as large as the genetic variance.

## Discussion

Heritabilities of health traits in different species are usually found to be low, which might reflect a large environmental influence or suboptimal recording of the traits. Moreover, many traits are expressed categorically, e.g. healthy vs. sick or clinical findings vs. no clinical findings, which together with the level of prevalence contribute to lower heritabilities on the observed scale of recording the traits. The data in this study showed that sum of clinical findings within type of examination produced heritabilities of up to 0.12 for PALP and 0.10 for HOOF findings. When defining PALP findings further into separate clinical signs and systemic locations, respectively, the heritability increased to 0.14 for effusions and to 0.13 for findings related to joints that were the dominantly occurring PALP findings [11]. For comparison, health and functional traits of dairy cattle, such as somatic cell count as indicator of mastitis, and female fertility, have mostly shown heritabilities of 0.01–0.14 [13]. Despite the low heritabilities the genetic variation *per se* has shown to be sizeable and has led to distinctly improved breeding programs [14]. This suggests good opportunities for similar population improvement in PALP and HOOF health of horses if including these health traits in the genetic evaluations and selection programs. However, due to the weak and non-significant genetic correlation between PALP and HOOF, both traits need to be selected for in order to improve both traits.

The estimated heritability of joint findings is equal to the previously estimated underlying quantitative heritability of OC overall, including fetlock-, tarsal- and patellar joint findings, in the SWB [7]. Possibly, affected horses might coincide to some extent in these two studies, where joint effusion could be an early symptom of OC. It might be speculated that the low heritabilities of other clinical signs and systemic locations in some cases might be influenced by relatively low prevalences. However, chronic joint lesions may also have a stronger genetic background compared to e.g. acute lesions that expectably are due to environmental effects to a larger extent. The estimated heritabilities of separate hoof characteristics in the SWB (0.02–0.11) are lower than the estimates from the Dutch warmblood studbook admission inspection of hoof related traits at between 0.12–0.27 [15] and of hoof characteristics in Icelandic horses at 0.45 [16]. Variations may be due to population differences in hoof variation or included hoof traits in each estimate. Possibly, Swedish veterinarians might need to be encouraged to focus more on the hoof examination than has previously been practiced. There is also a possibility that hoof characteristics are influenced by limb conformation and thus limb loading, as well as environmental factors regarding shoeing intervals etc. which may or may not be different in different populations.

The correlation between HOOF and LOCO/PALP were positive but non-significant. The possible influence of hoof quality on health status may need to be studied for more specific traits to show any significance. Furthermore, development of secondary effects of poor hoof status may increase at an older age when horses have been exposed to higher intensity of training for a longer period. It could be speculated whether the high (but non-significant) genetic correlation of MED and LOCO might be a result of an overall predisposition to impaired health that affects both results. Most phenotypic correlations were weaker than the corresponding genetic correlations, which is expected, however, this was not seen for the correlation between MED and H1, suggesting environmentally influenced factors within MED to largely influence the H1 score.

### Recording regime

Generally, summed findings for type of examination produced higher heritabilities compared to overall scores given by the examining veterinarian. In case of the H2 score the heritability was identical to the earlier estimate of the same trait by Wallin *et al.*[10]. The higher heritabilities for type of examination might be due to a higher specificity of recording, fairly standardised practice of scoring, and a less subjective evaluation, compared to assessed overall scores where several types of examinations were combined. The low heritability estimate of MED may be due to inclusion of a wide spread of non-related clinical findings, commonly represented of e.g. scars and skin wounds that are only temporarily present and highly dependent on environmental exposure. Such traits may be present in all horses at some point in life but are merely assigned to chance if affected at a particular day or not.

The recording of LOCO was almost as specific as PALP regarding recording regime and with an identical grading scale used. Still LOCO produced distinctly lower heritability estimates. Thus, LOCO results might be more influenced by environmental factors, e.g. training intensity as a cause of unsoundness. Further, differences in regime of examination between veterinarians might contribute to the environmental variation. However, R^2^ values revealed that fixed effects of event and gender explained only half the variation of LOCO, compared to other types of examination, suggesting LOCO to be less influenced by fixed effects than other types of examination. Possibly, efforts towards harmonisation of LOCO assessments have been more successful compared to other health examinations. This would imply that non-systematic factors, such as training intensity, stand for a substantial part of the variation in LOCO results. However, other studies have shown that the agreement between experienced veterinarians examining the same horses with mild lameness is low and that the rate of provocation during flexion tests, i.e. applied force and time, varies between veterinarians and has a significant effect on the results [17,18]. Accordingly, whether flexion tests are valid in the absence of standardised regimes has been debated. The heritability of lameness in 265 Norwegian Standardbred trotters was estimated to 0.25 (s.e. 0.21), when all horses were examined by the same veterinarian including flexion tests [19]. The estimate was based on a small number of horses and had a high standard error but might still suggest that the heritability of lameness in horses may be distinctly larger than those generated in the present study. Differences might be breed dependent, but could also reflect the importance of standardised examinations and recording regimes, perhaps aided by modern lameness diagnostics equipment, to obtain comparable information within a population. The lower heritability of hindlimb flexion test results compared to forelimbs is not due to differences in prevalence or effects on overall health. On the contrary hindlimb lesions are more common, and the effect on H2 scores was similar for flexion tests of both fore- and hindlimbs [11]. However, R^2^ values indicate that effects of event and gender had a somewhat larger impact on hindlimb flexion test results (0.16) compared to forelimbs (0.12), suggesting a larger variation among examiners for hindlimb flexion test results compared to forelimb results. Possibly, it is easier to correctly diagnose forelimb health status after flexion tests, due to differences in anatomy where present forelimb lesions are harder to compensate for compared to hindlimb lesions.

### Genetic vs. environmental effects on health status

The 4–5 times larger effect of event compared to the genetic variation, indicates that further harmonisation of examination regimes would be advantageous to the RHQT as well as for practicing veterinarians. The horse industry relies on similar examination results for e.g. sales and insurance purposes, and the results should be independent of choice of examiner. The event effect also accounts for time differences regarding development of veterinary care and use of limb correction regimes, and also, horse owner awareness and thus choice of horses entered. In the present study the effect of event was corrected for, which adjusts for the present veterinary and time differences.

The large genetic variation was demonstrated by calculated additive genetic coefficients of variation and the large differences between top and bottom stallions regarding offspring health status of PALP and HOOF.

### Factors contributing to genetic improvement of health traits

Prior to use in breeding, SWB stallions are examined for health status, including radiographic examination for OC and bone spavin, in connection to the stallion performance test. However, no routine genetic evaluation of health traits in the SWB has previously been practiced. Despite this absence, a consistent genetic improvement of health status, corresponding to one third of a genetic standard deviation, has occurred in the studied population between 1983 and 2001 for HOOF and PALP, respectively (Figure 3). Influencing factors for the genetic improvements may be the phenotypic stallion health inspections, and an indirect selection for longevity as life time performance is selected for. The trait for competition performance, included in the genetic evaluation, is based on accumulated competition results [20]. Thus, stallions with the highest performance results throughout several years obtain the best values. Another factor influencing genetic improvement for health might be that the RHQT health recordings, which have been conducted since 1973, to some extent have contributed with an informative description of individual horses’ health status to breeders. For further improvements, specific breeding values for orthopaedic health in stallions may be introduced as a complement to present breeding values for performance. Indexes could be produced either as separate evaluations of PALP and HOOF, or as a combined orthopaedic health index of PALP, HOOF and LOCO where the information from the H2 score also may be included. The ability for mares to produce healthy offspring is harder to correctly estimate as they produce less progeny at a slow rate in combination with a low heritability, resulting in low accuracy of breeding values. Nevertheless the role of mare health status should not be disregarded and in a BLUP animal model all information about relatives is also included, thus raising the accuracy in mare evaluations.

## Conclusion

Heritability estimates, results of genetic variation and differences in prevalence between sire progeny groups for PALP and HOOF clearly imply that health status in the SWB population can be improved by selection for health. The weak and non-significant correlation between PALP and HOOF suggests that both traits need to be selected for in practical breeding to improve both traits. Some genetic improvements over time have already been achieved, possibly due to regular stallion health inspections and an indirect selection for longevity through lifetime performance in sport. For further improvements stallion breeding values for health may be introduced, based on RHQT examinations, complementary to present breeding values for performance. Further harmonisation of regimes for veterinary examinations of young horses for important health traits should also be encouraged.

## Endnotes

^a^ DMU version 6 release 5, Research Center Folum, Tjele, Denmark.

^b^ SAS Institute Inc., Cary, NC, USA.

## Abbreviations

H1: Overall score, health examination 1 (medical and hooves); H2: Overall score, health examination 2 (orthopaedic); HOOF: Hoof examination; LOCO: Locomotion examination; MED: Medical examination; PALP: Palpatory orthopaedic examination; RHQT: Riding horse quality test; SWB: Swedish warmblood studbook.

## Competing interests

All authors declare that they have no competing interests.

## Authors’ contributions

All authors contributed to the planning, data evaluation and writing the article. LR and LJ executed the digitalisation of data used in the study. All authors read and approved the final manuscript.
